# Automated Recognition of Retinal Pigment Epithelium Cells on Limited Training Samples Using Neural Networks

**DOI:** 10.1167/tvst.9.2.31

**Published:** 2020-06-16

**Authors:** Qitong Gao, Ying Xu, Joshua Amason, Anna Loksztejn, Scott Cousins, Miroslav Pajic, Majda Hadziahmetovic

**Affiliations:** 1 Department of Electrical and Computer Engineering, Duke University, Durham, NC, USA; 2 Department of Ophthalmology, Duke University, Durham, NC, USA; 3 Department of Computer Science, Duke University, Durham, NC, USA

**Keywords:** retina, RPE, deep learning

## Abstract

**Purpose:**

To develop a neural network (NN)–based approach, with limited training resources, that identifies and counts the number of retinal pigment epithelium (RPE) cells in confocal microscopy images obtained from cell culture or mice RPE/choroid flat-mounts.

**Methods:**

Training and testing dataset contained two image types: wild-type mice RPE/choroid flat-mounts and ARPE 19 cells, stained for Rhodamine-phalloidin, and imaged with confocal microscopy. After image preprocessing for denoising and contrast adjustment, scale-invariant feature transform descriptors were used for feature extraction. Training labels were derived from cells in the original training images, annotated and converted to Gaussian density maps. NNs were trained using the set of training input features, such that the obtained NN models accurately predicted corresponding Gaussian density maps and thus accurately identifies/counts the cells in any such image.

**Results:**

Training and testing datasets contained 229 images from ARPE19 and 85 images from RPE/choroid flat-mounts. Within two data sets, 30% and 10% of the images, were selected for validation. We achieved 96.48% ± 6.56% and 96.88% ± 3.68% accuracy (95% CI), on ARPE19 and RPE/choroid flat-mounts.

**Conclusions:**

We developed an NN-based approach that can accurately estimate the number of RPE cells contained in confocal images. Our method achieved high accuracy with limited training images, proved that it can be effectively used on images with unclear and curvy boundaries, and outperformed existing relevant methods by decreasing prediction error and variance.

**Translational Relevance:**

This approach allows efficient and effective characterization of RPE pathology and furthermore allows the assessment of novel therapeutics.

## Introduction

As artificial intelligence-based techniques are getting more popular in ophthalmology, assessing retinal pathology using this approach has gained more attention.^1^ Screening, diagnosis, and treatment outcomes of major retinal diseases, including age-related macular degeneration (AMD), diabetic retinopathy (DR), and diabetic macular edema (DME), nowadays depend significantly on novel imaging technologies that are amenable to automatization. Machine learning (ML),^2^ a branch of AI, and the corresponding neural networks (NNs)^3^ have been integrated into the diagnosis of retinal diseases and have demonstrated utility to improve diagnostic efficiency and accuracy.^1^^,^^4^ For several years, convolutional neural networks (CNNs),^5^ a subclass of NNs, have been applied successfully in the detection and classification of retinal pathology.^6^^,^^7^

Unlike clinical research in ophthalmology, the use of NNs has attracted less attention in basic ophthalmic research. There is an unmet need for a universal, automated tool that facilitates the recognition of various retinal cell types. This type of innovation would make retinal basic research easier and would spare scientists from time-consuming, manual cell counting.

The retinal pigment epithelium cells are specialized (RPE), monolayer, hexagonal cells that play many roles crucial for retinal health.^8^^–^^11^ There are a variety of retinal disorders in which the primary site of pathogenesis is RPE, and AMD is the most prevalent one. Analysis of the RPE from different in vivo and in vitro models has provided us with valuable insight into the retinal pathology. Regardless of their shortcomings, work with ARPE-19 allows us to investigate the morphology and very complex dynamics of these cells. Analysis of the tissue obtained from the human donor eyes, or different animal models of retinal diseases, is still the principal way to investigate changes in morphology associated with different retinal diseases affecting the RPE.

In this study, we were focusing on the automated detection of RPE cells in an animal model (wild-type mice RPE/Choroid flat-mounts) and the cell culture (ARPE19). Although some existing approaches may be able to address this problem, they either require enormous training resources (i.e., a very large number of labeled images) or they cannot produce precise predictions due to limited expressiveness. Specifically, fully convolutional neural networks (CNNs), such as UNet,^12^ can be applied to mapping the input images to the corresponding feature maps, which are then post-processed to identify RPE cells. However, a substantial number of training images is required for the CNN to learn an accurate mapping, even with transfer learning.^13^ On the other hand, linear regression models, as proposed by Lempitsky et al.^14^ and Hoerl et al.,^15^ fail to capture the desired input-output relation accurately, as we show in the results section. To resolve these issues, we propose a novel approach to detect RPE cells effectively and precisely with limited training resources, but without compromising the expressiveness of the model. Moreover, we validate our approach with two different types of RPE cells – ARPE19 and wild-type mice RPE/Choroid flat-mounts. Specifically, following the proposed methodology, we trained two NN models on the two different types of RPE cell images. Finally, we demonstrated that our method outperforms the existing methods, proposed by Lempitsky et al. ^14^ and Hoerl et al.,^15^ on both types of RPE cell images.

The goal of this work was to address an urgent need in RPE cell analysis and develop an essential tool for future studies that rely on the retinal cell morphology to investigate the onset and progression of retinal diseases and response to the treatment. We introduce an NN-based approach, which can be used even with limited training data, to train an NN model with rich enough expressiveness, capable of successfully reconstructing the ground-truth cell density distribution; and thus automatically accurately identify and count the number of RPE cells.

## Methods

ARPE-19 cell lines in cell culture and RPE/Choroid flat mounts from wild-type mice (C57BL/6J) were used for the RPE cell count. Handling and staining procedures were described in Appendix A. All procedures were approved by the Institutional Animal Care and Use Committee of the Duke University and complied with the ARVO Statement for the Use of Animals in Ophthalmic and Vision Research.

### Fluorescence Microscopy

Fluorescence microscopy was performed using Nikon Eclipse 90i confocal microscope equipped with ×20 air objective. For the quantification, maximum intensity projection images were extracted from each z-stack using Fiji, an open-source image processing software 36. The number of RPE cells was counted using Fiji software within the corresponding area.

#### NN Model Design

**Figure 1.**
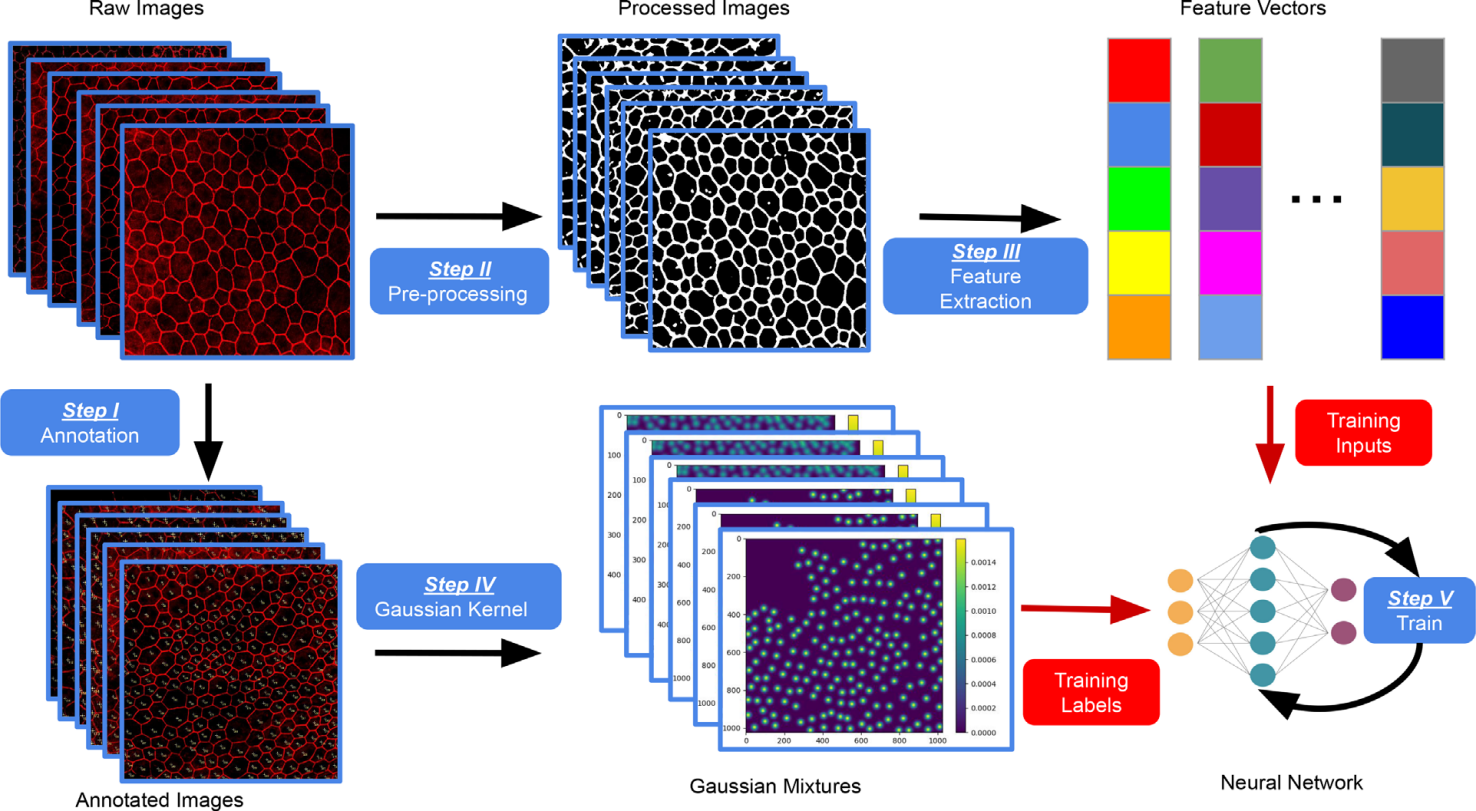
Overview of the presented neural network (NN) design methodology. After image preprocessing for denoising and contrast adjustment, scale-invariant feature transform descriptors were used for feature extraction. The set of training labels was derived from cells in the original training images, annotated, and converted to Gaussian density maps as a sum of fixed-variance Gaussians centered at each annotation. An NN was trained using the set of training input features and labels such that the obtained NN model accurately predicts the corresponding Gaussian density maps and thus identifies/counts the cells for any image.

Our NN-model design method, illustrated in Figure 1, contained the following three phases: (1) image annotation (Step I) and preprocessing (Step II); (2) forming the training dataset—i.e., converting images into suitable image features (Step III) and training labels (Step IV); and (3) NN training through optimization—i.e., stochastic gradient descent (Step V).

**Figure 2.**
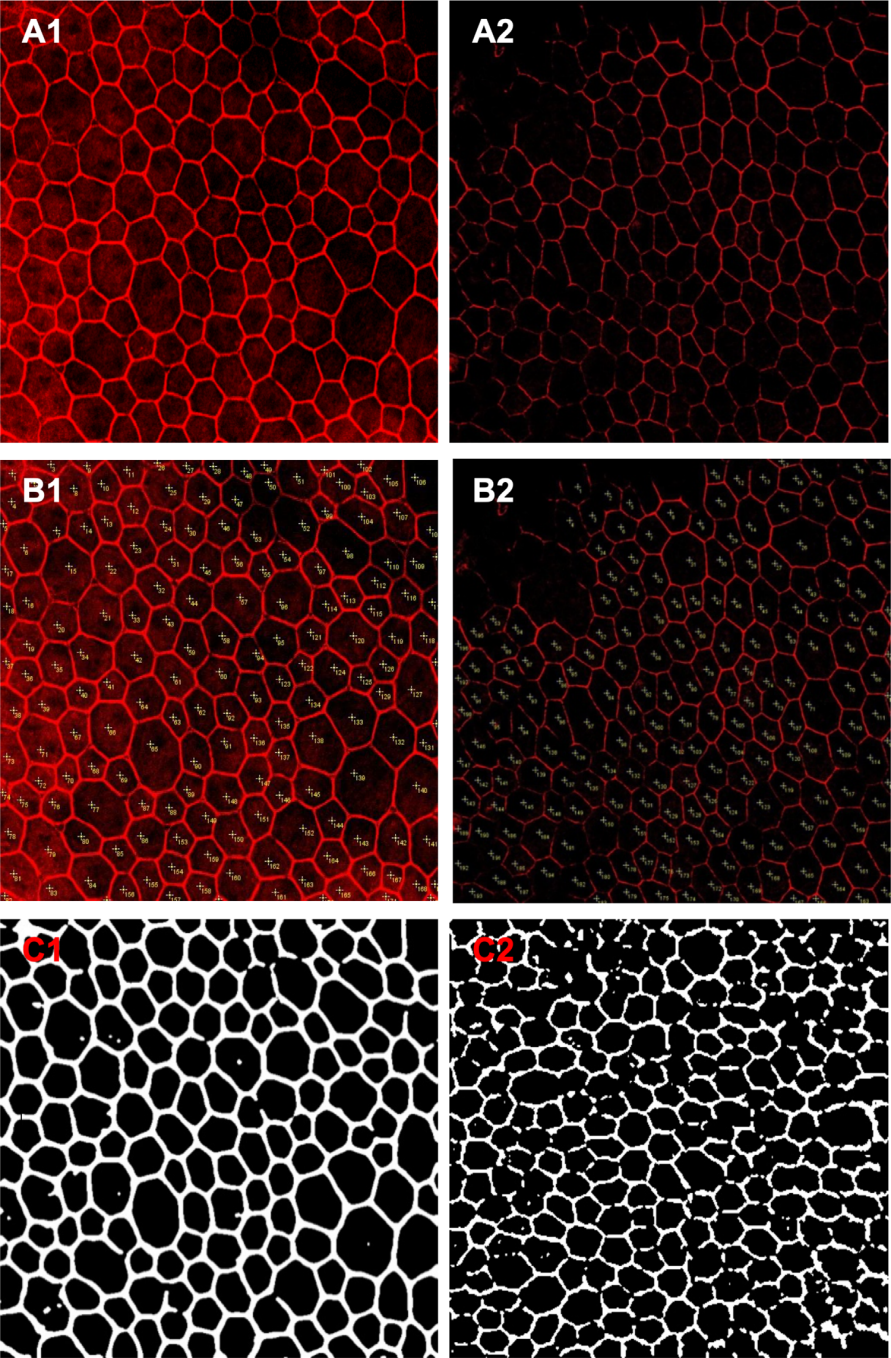
Image annotation and preprocessing. Example of exposed and unexposed raw images (A1 and A2); annotated exposed and unexposed images (B1 and B2); processed exposed and unexposed images (C1 and C2).

**Image** **Annotation** **and** **Preprocessing** **(Steps I** **and** **II in** **Fig. 1****).** We started from a training set, containing either raw RGB images of RPE/Choroid flat-mounts or ARPE19 cells, as shown in Figure 2 (A1 and A2). In Step I, these images were first annotated by humans; specifically, each cell in a raw image was labeled by a cross within the cell boundary, as in Figure 2 (B1 and B2). Furthermore, in Step II, each image was automatically converted into a greyscale image and processed (i.e., filtered) to adjust contrast (details are presented in Appendix B); this allowed for the use of images obtained under different exposure conditions. For example, Figure 2 shows the results from this method on a sufficiently exposed (C1) and underexposed image (C2).

**Forming the Training Dataset.** In the next step (Step III), we derived a suitable set of image features that were to be used as training inputs for the learning algorithm. We applied a common computer vision approach as previously described.^14^^,^^16^^,^^17^ Specifically, we used the scale-invariant feature transform (SIFT) to convert each grayscale image *I_k_* into a set of feature vectors $f_{I_{k}}^{j}$, where the number of vectors is equal to the number of pixels in the initial grayscale image; and thus such vectors could capture a large number of hidden features that are not explicitly shown in the image. Note that the SIFT descriptors are used to replace the convolutional layers in CNNs. Although both can be used to extract features of images, CNNs required substantial training data,^13^ whereas SIFT descriptors did not need to be trained.

**Figure 3.**
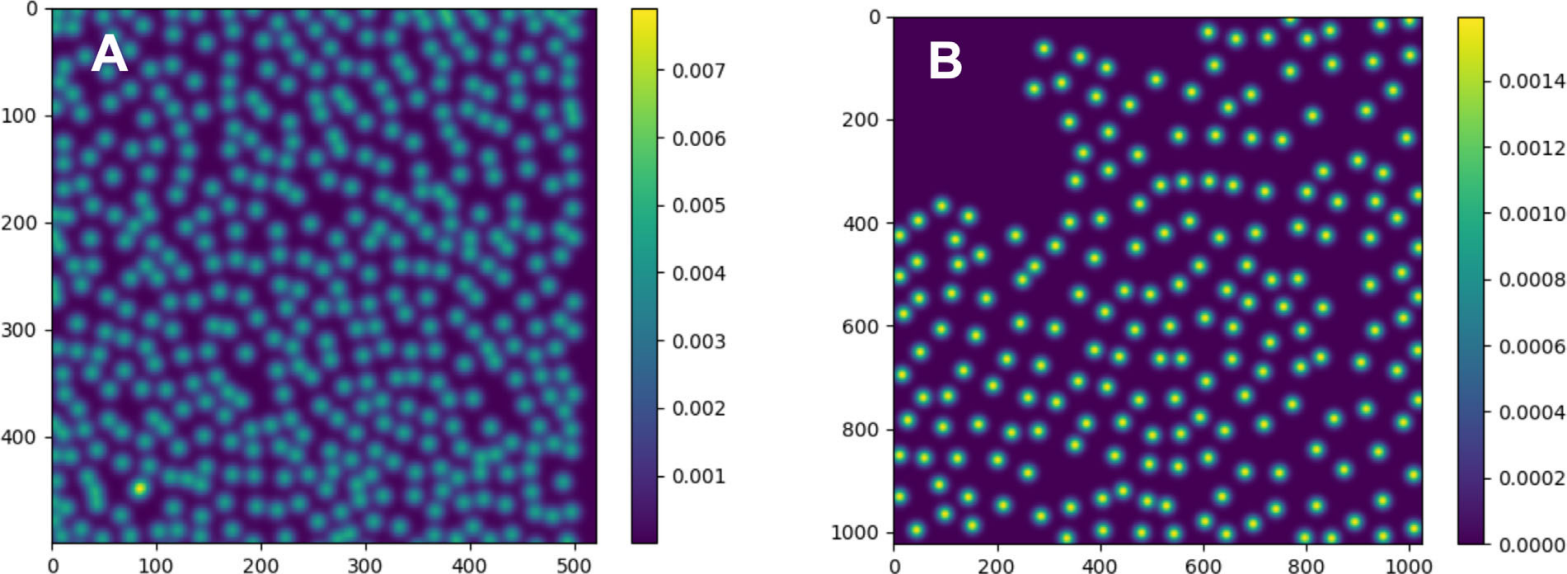
GMs of exposed and underexposed raw images (A and B).

Furthermore, to obtain suitable training labels for the annotated images, (in Step IV) for each annotated image, we generated normalized two-dimensional discrete Gaussian kernels centered at every annotated cell position in an image (i.e., crosses in Figs. 2B1 and 2B2); the covariance of these kernels was chosen such that the center part of each resulting Gaussian was included in the cell. This way we formed a Gaussian mixture (GM) $s_{I_{k}}$, for each image *I_k_* from the annotated image set, as illustrated in Figure 3.

**NN** **Training.** For our NN model, we used a network with two fully connected layers, each containing 2000 neurons, where all the hidden layers use the rectified linear activation whereas the output layer uses linear activation functions. The goal of the NN training was to find parameters of the NN (denoted by θ), such that the functionality of the NN *F*(*U_I_*; θ) closely enough approximates the GMs for all images; here, we denoted by *F*(*U_I_*; θ) the output of the NN with parameters (i.e., weights) θ, and input features *U_I_* obtained from the image *I* in Step III. (A formal [mathematic] review of the NN, as well as the input-output relation, is discussed in Appendix C.) Specifically, in our case, the inputs to the NN were the feature vectors $f_{I_{k}}^{j}\;$of each training images *I_k_*, whereas the outputs should closely approximate the corresponding GM $s_{I_{k}}$. To achieve this, we applied the stochastic gradient descent method^18^^–^^20^ to minimize the average approximation error on the training set with *N* images—i.e., we used the following loss function during training:

$$\min_{\theta }J\left(\theta \right)=\frac{1}{N}\sum_{k=1}^{N}{\left[F\left({U_{I}}_{k};\theta \right)-\;s_{I_{k}}\right]}^{2}.$$

Note that in our approach the training inputs were the feature vectors instead of entire images as was in the study by Xie et al.^16^ Consequently, a small training dataset that consists of only a few images could be sufficient to train the NN because of the fact that each image *I_k_* was converted to a set of feature vectors, whose number was equal to the number of pixels in the initial image.

#### Performance Metrics

We considered the average error rate (AER) to evaluate the performance of the trained NN on available set of *M* test images, which was defined as

$$AER=\frac{1}{M}\sum_{t=1}^{M}\frac{\left|F\left({U_{I}}_{t};\theta \right)-\;s_{t}^{*}\right|}{s_{t}^{*}}\times 100\%.$$

Here, *F*(*U_I_*_*t*_; θ) was the number of cells estimated by the trained NN with weights θ for the input features coming from a test image *I_t_*, whereas $s_{t}^{*}\;$was the ground-truth number of cells contained in *I_t_*.

We also defined the accuracy (ACC) as

$$\begin{array}{ccc} ACC & = & 1-AER=\left(1-\frac{1}{M}\sum_{t=1}^{M}\frac{\left|F\left({U_{I}}_{t};\theta \right)-\;s_{t}^{*}\right|}{s_{t}^{*}}\right)\\ & & \times 100\%. \end{array}$$

#### Experimental Setup

**Figure 4.**
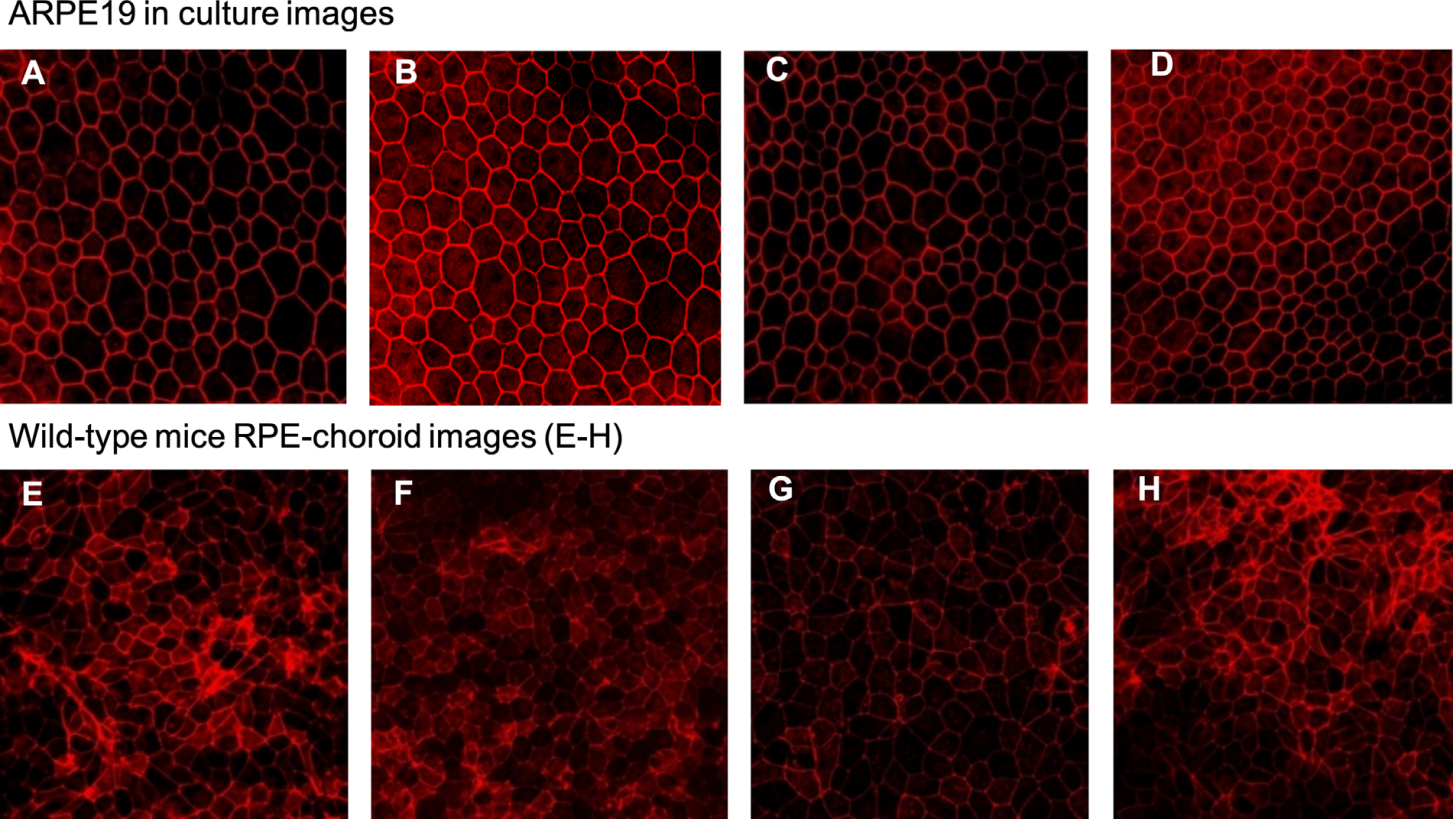
Photomicrographs show uneven image quality in cell culture (A–D) and animal tissue (E–H).

The dataset we used to train and validate our model contained two different types of images: wild-type mice RPE/choroid flat-mounts and ARPE 19 cells in culture, stained for rhodamine-phalloidin and imaged with confocal microscopy, as previously described. Besides the tissue nature and preparation, these two types of images have significant dissimilarities, as shown in Figure 4. Hence, we trained two NNs using the previously discussed methodology, one for wild-type mice RPE/Choroid flat-mounts and the other for ARPE 19 cells in culture.

**Figure 5.**
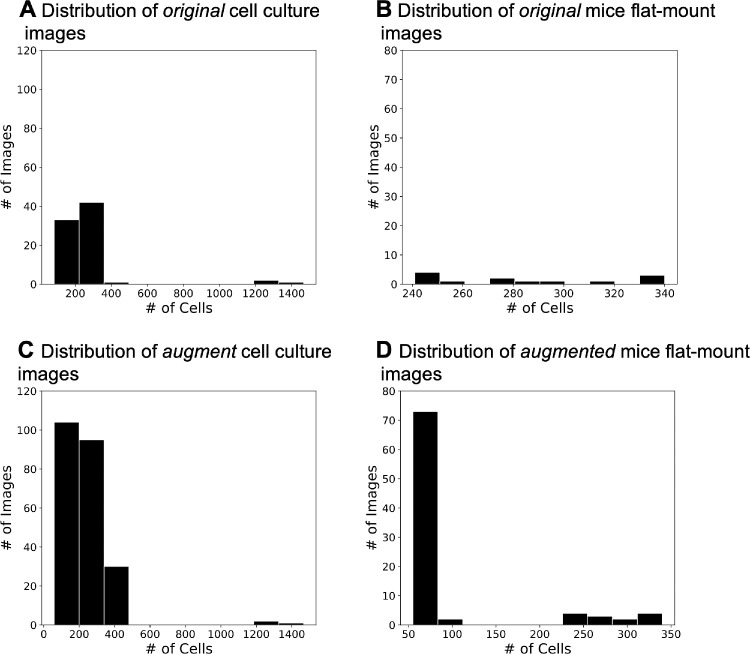
The histograms show the numbers of the *original* cell culture and *original* mice images before (A, B) and after augmentation (C, D). The x-axis corresponds to the number of cells that are contained in an image, and y-axis captures the number of images contained in the dataset.

The original cell culture dataset (OCCD) and original mice flat-mount dataset (OMFD) contained 79 from cell culture and 13 mice flat-mount images respectively (Table 1, Figs. 5 A and 5B). We augmented both datasets by randomly cropping 15 sub-images out of the original images, containing greater than 350 cells for cell culture and 300 cells for mice flat-mounts. The resulting augmented cell culture dataset (ACCD) and augmented mice flat-mount dataset (AMFD) contained 229 and 88 images, respectively (Figs. 5C and 5D).

**Table 1.** Distribution of Training-Testing Division

	Overall Augmented Dataset				Train				Test			
		Original	From			From	From			From	From	
Type	Name	images	cropping	Total	Name	original	cropping	Total	Name	original	cropping	Total
Cell	ACCD	79	150	229	C-Tr	61	100	161	C-Te	18	50	68
Mice	AMFD	13	72	85	M-Tr	10	68	76	M-Te	3	6	9

The statistics of the ACCD and the AMFD are shown in the first column, C-Tr and M-Tr in the second column, and C-Te and M-Te in the third column, where the number of original and cropped images contained in each set is shown as subcolumns within each column.

Furthermore, we divided the ACCD and AMFD into training sets and testing set for cell and mice images, respectively. Specifically, from the ACCD we randomly selected 70% of images to constitute the *cell image training set* (C-Tr), with the remaining 30% used as the *cell image testing set* (C-Te). From the AMFD, we randomly selected 90% of images to form the *mice image training set* (M-Tr), with 10% forming the *mice image testing set* (M-Te). This increased the ratio of training images to improve training performance due to the small size of the AMFD. This has been captured in Table 1. Then, two NNs were trained to count the images in ACCD and AMFD, respectively. Specifically, the first NN was trained with images in C-Tr and evaluated with C-Te, and the second NN was trained with M-Tr and evaluated with M-Te.

## Results

Our training results were noninferior to chosen baseline approaches. We compared our algorithm to two baseline approaches: (1) the method proposed by Arteta et al.,^21^ where linear ridge regression (LRR),^20^ instead of NNs, was used to map the SIFT descriptors to the GMs—we referred to it as LRR method, and (2) a widely used counting method by Lempitsky and Zisserman from the Oxford, Visual Geometry Group,^14^ which we referred to as the LZ method. A brief introduction of the two baseline approaches is presented in Appendix D. Then, two NNs were trained to map the input images, from ACCD and AMFD, respectively, to corresponding GM densities. The performance was quantified by the AER and ACC defined in (2) and (3), respectively.

**Figure 6.**
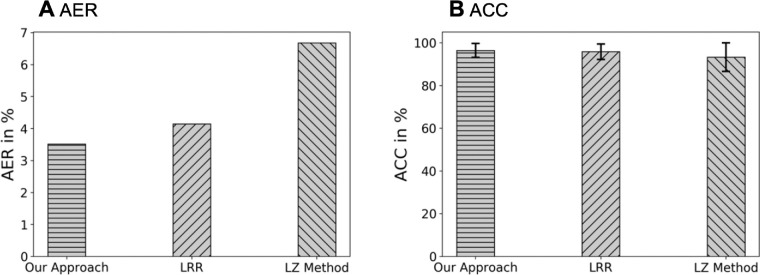
The AER and ACC on all (randomly cropped images + the originals) cell images in the C-Te, comparing three different approaches: our approach, LRR15 and the LZ method.^14^ The AERs are 3.52%, 4.15% and 6.68%, respectively. The ACCs with 95% confidence intervals are 96.48% ± 6.56%, 95.85% ± 7.30%, and 93.32% ± 13.44%, respectively.

We first evaluated the performance of our approach on *all* images (randomly cropped images + the originals) from the C-Te set. The AER over *all* images was 3.52% for our approach, a significant improvement over 4.15% and 6.68% for the LRR and LZ, respectively (Fig. 6A). The ACC over *all* images in the C-Te test was 96.48%, 95.85%, and 93.32% with standard deviation of 3.28%, 3.65% and 6.72% while training with our approach compared to LRR and LZ, respectively (Fig. 6B).

**Figure 7.**
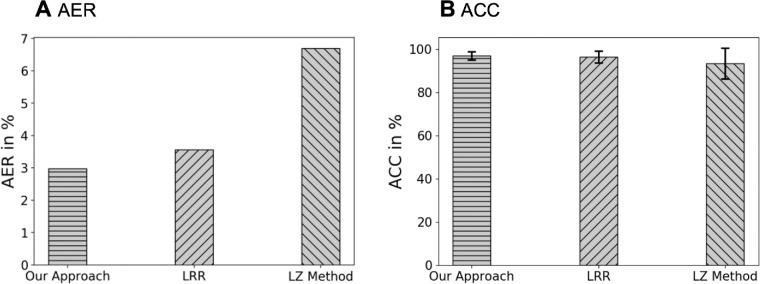
The AER and ACC on original cell images in the C-Te, comparing three different approaches: our approach, LRR^15^ and the LZ method.^1^^4^ The AERs are 2.97%, 3.56%, and 6.70%, respectively. The ACCs with 95% confidence intervals are 97.03% ± 3.88%, 96.44% ± 4.24%, and 93.30% ± 15.64%, respectively.

We also compared performance across the three algorithms on the *original* images in the C-Te set. Specifically, 2.97%, 3.56%, and 6.70% of AER was attained by our algorithm, LRR and LZ, respectively (Fig. 7A). Moreover, 97.03%, 96.44%, and 93.3% of average ACC, with standard deviation of 1.94%, 2.12%, and 7.82% was achieved by our algorithm, LRR and LZ, respectively (Fig. 7B).

**Figure 8.**
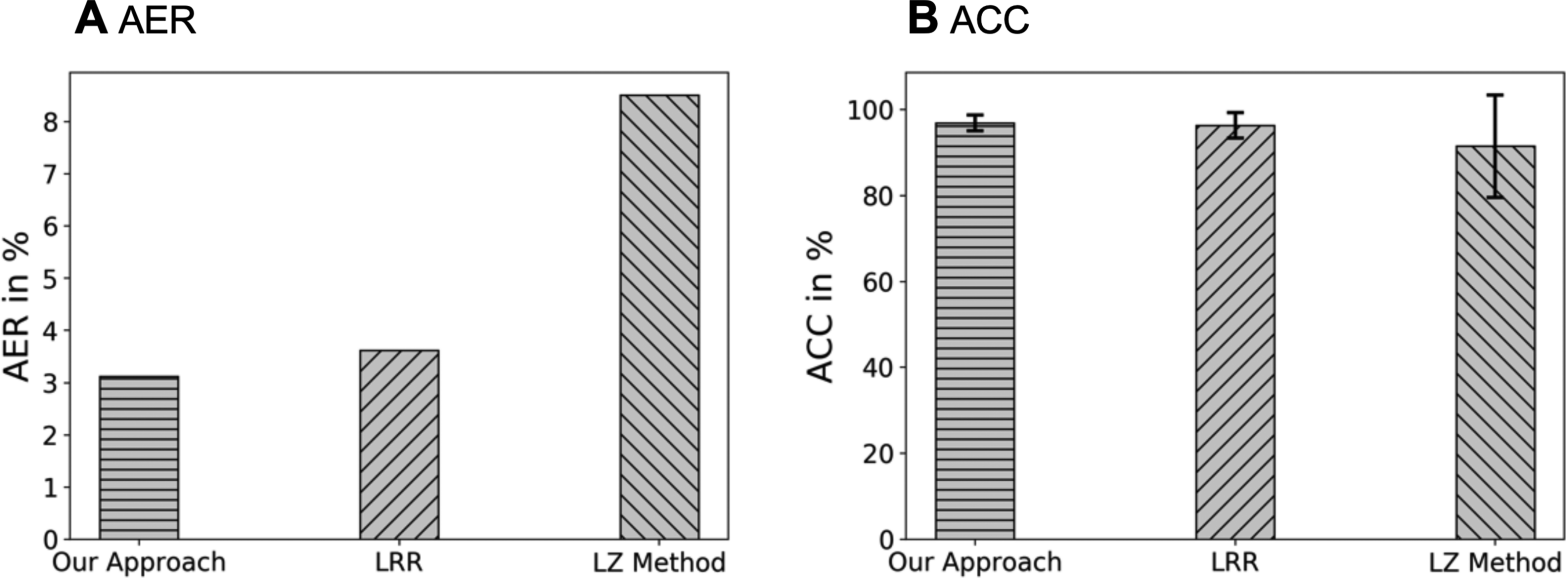
The AER and ACC on all mice flat-mount images in the M-Te, comparing three different approaches: our approach, LRR^15^ and the LZ method.^1^^4^ The AERs are 3.12%, 3.62% and 8.51%, respectively. The ACCs with 95% confidence intervals are 96.88% ± 3.68%, 96.38% ± 5.88%, and 91.49% ± 23.88%, respectively.

We also evaluated the performance of our approach and the two baseline approaches on the M-Te dataset. Specifically, our approach attained a 3.12% AER, whereas the LRR and LZ methods obtained AER of 3.62% and 8.51%, respectively (Fig. 8A). Moreover, our approach achieved ACC of 96.88% with a standard deviation of 1.84%, whereas the LRR and LZ methods had ACC of 96.38% and 91.49%, respectively, with standard deviation of 2.94% and 11.94%, respectively (Fig. 8B).

We summarized the results shown above in Table 2 and observed that our approach outperforms both the LRR and the LZ methods. Specifically, when evaluating with the *entire* C-Te test dataset, our algorithm decreased the AER by 15.18% and 47.31% compared with the two baseline methods. With the *entire* M-Te test dataset, our approach decreased the AER by 13.81% and 63.34% compared with the two baselines, respectively. Our algorithm also achieved significantly lower standard deviation than the two baseline methods, indicating that the results provided by our approach had lower variance and higher confidence levels with limited training images, where the training sets C-Tr and M-Tr only contained 161 and 76 images, respectively. On the other hand, we tested our algorithm on the *original* images in the C-Te test dataset to validate its performance on images that were not augmented by cropping (the third column of Table 2). Our approach again exhibited the lowest AER and standard deviation in both cases.

**Table 2.** Testing Results Summary

	Entire C-Te			Entire M-Te			Only original		
	Dataset			Dataset			images in C-Te Dataset		
	Average	Average		Average	Average		Average	Average	
Approach	SER/%	ACC/%	STD/%	SER/%	ACC/%	STD/%	SER/%	ACC/%	STD/%
**1. Our Approach**	**3.52**	**96.48**	**3.28**	**3.12**	**96.88**	**1.84**	**2.97**	**97.03**	**1.94**
2. LRR	4.15	95.85	3.65	3.62	96.38	2.94	3.56	96.44	2.12
3. LZ Method	6.68	93.32	6.72	8.51	91.49	11.94	6.70	93.30	7.82

Our approach achieved the highest ACC (or lowest SER) across the three test sets. Moreover, the predictions from the proposed method have the lowest STD, which indicates higher confidence level.

We concluded that our method counted the number of cells contained in cell culture and flat-mount images effectively and accurately. Furthermore, considering that both the OCD and OMD microscopy images were of uneven qualities (as shown in Fig. 4), the results above demonstrated that our approach was capable of accurately counting various types of input images.

## Discussion

There is an unmet need, not only in ophthalmic basic research but also generally for an automated cell counting tool that can help researchers with information processing and a better understanding of disease processes (characterization, progression, response to the treatment, etc.). Herein, we proposed an NN-based approach to recognize and count RPE cells in images obtained by confocal fluorescence microscopy from two different specimens: ARPE-19 cell culture and RPE/Choroid flat-mounts. Compared to the baseline approaches proposed by Lempitsky et al.^14^ and Hoerl et al.,^15^ our method achieved high accuracy even with limited training datasets and without compromising the expressiveness of the learning model. The CNN-based counting approach proposed by Xie et al.^16^ and Lu et al.^34^ cannot be applied in our instance because in this case the counting was performed only through the nucleus channel. Because of the nature of RPE cells, the number of nuclei is not always in clear correlation to the number of cells,^22^^,^^23^ and that is why we wanted to avoid this approach. The image preprocessing procedure that we designed can effectively standardize the various input images taken under different lighting and exposure conditions, which then ensures the performance of the following learning step. As shown in our results, the presented preprocessing and feature extraction method combined even with a simple linear estimator such as LRR (i.e., without the use of NN models), outperformed the LZ method by Oxford, Visual Geometry Group.^14^ When combined with an ML approach, our methodology resulted in a highly-accurately NN estimation model of the number of cells in used images.

Prior work done by Lempisnky et al.^14^ solved the density estimation problem using convex quadratic programming. However, this approach is only suitable with problems that can induce convex loss functions. Instead, we explored the use of ML and introduced a NN-based method that can be used to optimize non-convex and non-linear loss functions by performing stochastic gradient descent steps.^24^^–^^26^ Additionally, a CNN method proposed by Xie et al.^16^ requires a large number of training images. In contrast, we used the feature vectors, extracted from the original images as inputs to NN. In our method, we first applied SIFT^27^^,^^28^ to transform each pixel in a cell image to a corresponding feature vector, and then we used these feature vectors to train NNs to capture the underlying relations between the input vectors and the ground-truth cell density distributions. Combining the SIFT descriptors with multilayer perceptron NNs allowed us to accomplish the tasks that usually require deployment of CNNs with significantly smaller training dataset. Furthermore, in this work, SIFT descriptors, instead of the convolutional layers in the CNNs, were used to extract feature encodings from input images, and the training performance was no longer correlated with the size of training datasets. Hence, transfer learning was not necessary to be applied, because it is usually used to warm-start the training of the convolutional parts in the CNNs; on the other hand, in this work we used SIFT descriptors which can extract feature vectors without training. Considering the broad applications of SIFT descriptors in robotic vision, 3D modeling and gesture recognition,^29^^–^^31^ our approach can be generalized to solve other open problems related to medical images analysis by adjusting the SIFT descriptors to extracting features for specific types of images, along with designing suitable NN architectures.

Automated image segmentation is a critical step toward achieving a precise quantitative evaluation of disease states with different imaging techniques. CNNs proposed by LeCun et al.^5^ has been proved successful in extracting hidden features of image data^32^^–^^35^ and therefore can be applied to cell-counting problems. However, a great amount of training data is necessary to train a CNN. Moreover, because of regulatory constraints and privacy concerns, access to patient data is limited, and, as a result, CNN's may not perform well on the raw patient data. To resolve this issue, Xie et al.^16^ generated synthetic data^36^ to train the networks and use real images for fine-tuning. Lu et al.^37^ proposed a generic matching network with an adapter that customizes the network to any class of object by few-shot learning to perform training on small datasets. In addition, Chiu et al.^38^ proposed a cell boundary segmentation method using dynamic programming and graph theory, where one of the applications is cell counting. However, this approach can only segment the cells with *convex shape* boundaries. In contrast, our approach does not limit the cell shape of the input microscopy images. Furthermore, our method can accurately count images with unclear and curvy boundaries, e.g. images from OMFD which are shown in Figures 4E to 4H.

## Conclusion

In this work, we introduced a learning-based methodology to develop NN-based models that accurately estimate the number of RPE cells contained in images obtained from cell culture and mice flat-mounts. Moreover, we have presented an image preprocessing and data augmentation methods to form sufficient training images and improve the accuracy of the learning algorithm, even when a small number of training images were available. We have shown that our approach outperformed relevant methods by decreasing prediction error and variance significantly. This methodology used on large data set will be time-saving and possibly more precise and will allow for better characterization of diseases involving the RPE. The largest translation relevance of this approach will be the evaluation of novel therapeutics on the improvement of RPE health.

## Supplementary Material

Supplement 1

## Appendix A. RPE Cell Imaging Procedure

In this appendix, we introduce the procedure of preparing images that are used to train the NN models.

### Cell Culture

ARPE-19 cells were obtained from American Type Culture Collection (Manassas, VA, USA) and grown to confluence in Dulbecco's modified Eagle's F12 medium (DMEM/F-12; Gibco 11039-021) supplemented with 10% fetal bovine serum (FBS), 100 U/mL penicillin/streptomycin, and 0.38% Na_2_CO_3_ in a 5% CO_2_ humidified air incubator at 37°C. For experiments, cells were split and plated at sub-confluent density in six-well trans-well plates (Sigma CLS3450; Sigma-Aldrich, St. Louis, MO, USA) coated with 0.5 mg/mL collagen IV (Sigma C5533; Sigma-Aldrich), 5 mg/mL fibronectin (Sigma F0895; Sigma-Aldrich), and 50 ug/mL Laminin (Sigma L6274; Sigma-Aldrich). Cells were maintained in medium containing 5% FBS until fully differentiated and melanized (>8 weeks). To prepare samples for imaging, cells were fixed first in cold 4% paraformaldehyde (PFA) for 0.5 hour. Polyester membranes with bound cells were then placed on the glass microscopy slides. After the brief permeabilization in PBT buffer (PBS + 0.1% Triton × 100 + 0.5% bovine serum albumin), cells were then blocked using 10% normal donkey serum in PBT for 1 hour at room temperature. Samples were then incubated with 1:500 rhodamine-phalloidin (ThermoFisher Scientific R415; ThermoFisher Scientific, Waltham, MA, USA) overnight at 4°C, followed by nuclei staining with 1:5000 Hoechst 33258 (ThermoFisher Scientific H3569) solution in water. Fluoromount-G (ThermoFisher Scientific 00-4958-02), a clear liquid medium, was used to mount slides.

### Mice

BALBC and C57BL/6J wild type (WT) mice, 4 months of age or older, were obtained from The Jackson Laboratory (Bar Harbor, ME, USA). All procedures were approved by the Institutional Animal Care and Use Committee of the Duke University and complied with the ARVO Statement for the Use of Animals in Ophthalmic and Vision Research.

### RPE-choroid flat-mount Immunofluorescence

RPE-choroid flat-mounts were generated by removing the anterior segment and the neurosensory retina from the eye, followed by flattening of the eye cups on a glass slide with four relaxing incisions. The eye cups were then fixed first in cold 4% PFA for 0.5 hour, permeabilized briefly in PBST buffer (PBS + 0.1% Triton × 100) and blocked using 5% normal donkey serum in PBST for 1 hour at room temperature. Staining with rhodamine-phalloidin and Hoechst 33258 was performed according to protocol described for ARPE19 cells.

## Appendix B. Filtering of the Grayscale Images

The filtering procedure for the grayscale images, which were automatically obtained from RGB raw images is designed as follows. To process the images with high exposure, which have clear cell boundaries but significant noise in the background, we first applied erosion^29^ to shrink white noise; however, the cell edges may also fade as a side effect. Thus, to compensate for this, we binarized all the values to augment the cell boundaries (with a threshold equal to 0.5). Finally, we used a 3 × 3 kernel filter to further reduce the background noise. Specifically, for any pixels $p_{I_{k}}$ in a greyscale image *I_k_*, if the average of the surrounding pixels of $p_{I_{k}}$ is smaller than 0.5, we sorted the array that contains $p_{I_{k}}$and its surrounding pixels and assign the smallest value that is greater than 0.5 as the new value of $p_{I_{k}}$. Otherwise, we assigned the largest value that is smaller than 0.5 as the new value of $p_{I_{k}}$. In Supplementary Figure S1, we illustrate how two example pixels, *p*_1_ < 0.5 and *p*_2_ > 0.5,  are processed by the above filter and show that the proposed filter further reduces noise and augment dim boundaries.

## Appendix C. Input-Output Relation of the NN

An NN is a computational model that can be used to approximate an unknown mapping between the input space $X_{i}\subset R^{m}$ and the target space $X\subset R^{n}\;$, which satisfies some desired properties. Its structure can be decomposed layer by layer into matrix and function operations as

$$O_{i}=\phi_{i}\left(\theta_{i}O_{i-1}+b_{i}\right),\;i\in \left[1,l\right],$$

where *O_i_* is the output of the *i*th layer, φ_*i*_ is the activation function, θ_*i*_ is the weight matrix, *b_i_* is the bias vector and *l* is the total number of layers. Particularly we define *O*_1_ = φ_1_(θ_1_*U* + *b*_1_), where *U* denotes the input to the NN, and *O_l_* as the output from the NN. As a result, the relation between the output *O_l_* and the input *U* can be derived as a function *F* , i.e.,

$$\begin{array}{ccc} O_{l} & = & \phi_{l}(\theta_{l}\left(\phi_{l-1}\left(\theta_{l-1}\left(\cdots \phi_{1}\left(\theta_{1}U+b_{1}\right)\cdots \right)+b_{l-1}\right)\right)\\ & & +b_{l})=F\left(U;\theta \right),\;\;\; \end{array}$$

where θ = {θ_1_,θ_2_,…, θ_*l*_} represents the weights.

In our NN model, for any image *I* and its corresponding feature vectors $f_{I}^{1},\;f_{I}^{2},...,f_{I}^{m\times n}$, where *m* × *n* is the number of pixels in the image, without loss of generality, we denote

$$\begin{array}{c} F\left(I;\theta \right)=\sum_{i=1}^{m\times n}F\left(f_{I}^{i};\theta \right). \end{array}$$

## Appendix D. Overview of the Baseline Methods

The LRR model^21^ optimizes a linear model with weights *w* to minimize the loss *J_LRR_*(*w*), i.e.,

$$\min_{w}J_{LRR}\left(w\right)={\left|U_{I_{k}}w-s_{I_{k}}\right|}^{2}+\lambda {\left|w\right|}^{2},$$

where λ is the normalizing constant and $U_{I_{k}}$ and $s_{I_{k}}$ represent the matrix of feature vectors and the GM density, respectively as in (1).

The LZ method also proposes to optimize a linear model, with weights *w*, but minimizing a different loss function *J_LW_*(*w*), i.e.,

$$\min_{w}J_{LW}\left(w\right)=\sum_{k=1}^{n}D\left(U_{I_{k}}w,\;s_{I_{k}}\right)+\lambda {\left|w\right|}^{2},$$

where $U_{I_{k}}$ is the matrix of feature vectors, $s_{I_{k}}$ is the GM density, and *D*(·, ·) represents the Maximum Excess over SubArrays (MESA) distance function as defined in Lempitsky and Zisserman.^14^ Note that the $U_{I_{k}}$ and $s_{I_{k}}$ in (6) and (7) refer to the input features and the GM, respectively, as in (1). The *w* refers to the weights of the linear models, whereas in (1) the weight of the NN is denoted by θ to differentiate the linearities among models.
