# Supplementary material for: Automated Recognition of Retinal Pigment Epithelium Cells on Limited Training Samples Using Neural Networks
Source: Transl Vis Sci Technol. 2020 Jun 16;9(2):31. doi: 10.1167/tvst.9.2.31 (PMC7414692; doi:10.1167/tvst.9.2.31)
Supplement: Supplement 1 [file tvst-9-2-31_s001.pdf]

Supplemental Figure 1. Graphical description of self-designed filter

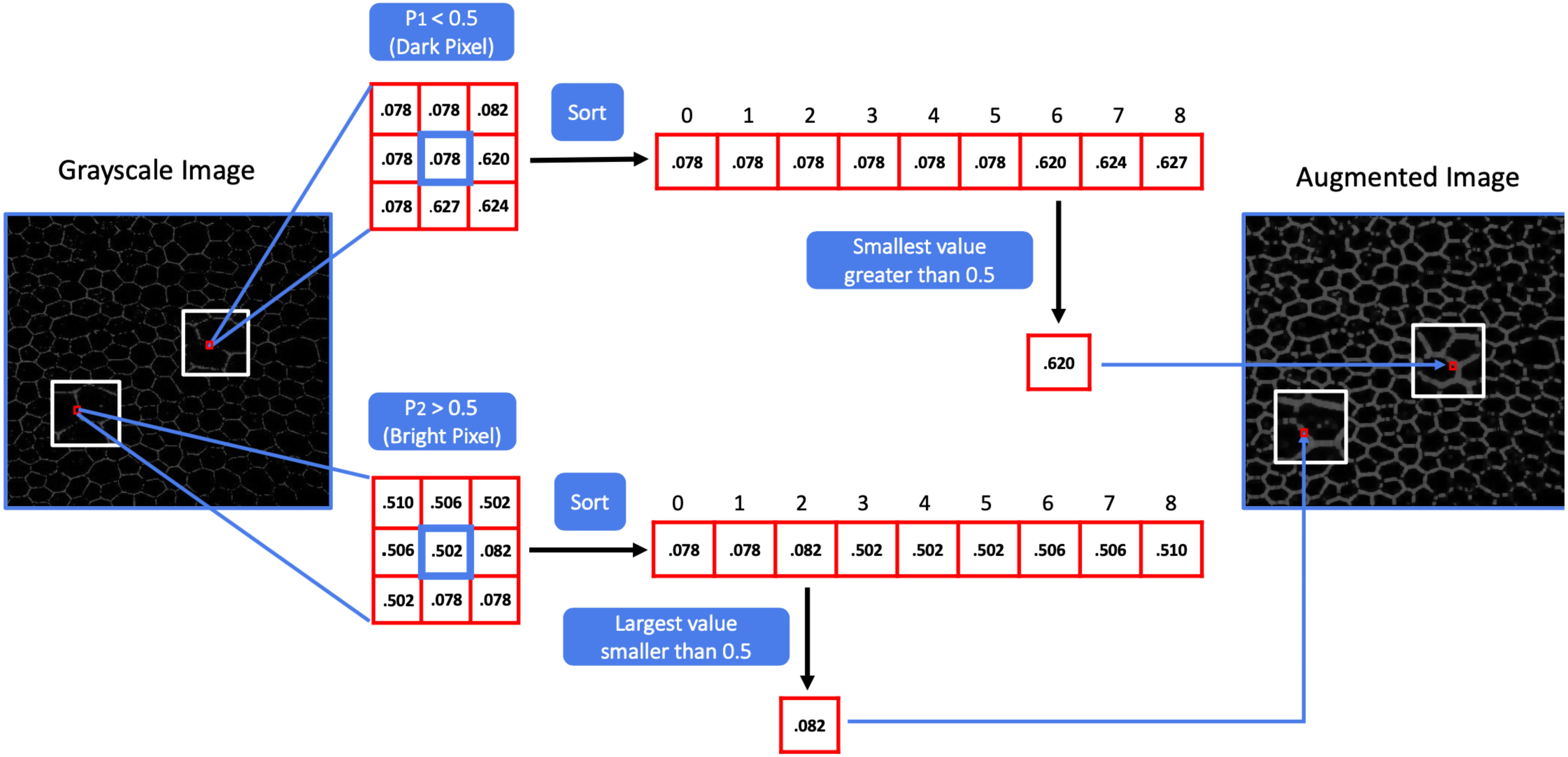

Supplemental Figure 1: Filtering for noise reduction. This figure shows how two pixels, $p_1 < 0.5$ and $p_2 > 0.5$, are processed by the proposed filter. For p_1 (i.e. dark pixel), the sorted array contains pixels $\{20, 20, 20, 20, 20, 21, 158, 159, 160\}$ and 158 is selected to be the new p_1. For p_2 (i.e. bright pixel), the sorted array contains pixels $\{20, 20, 21, 128, 128, 128, 129, 129, 130\}$ and 21 is selected to be the new p_2.
